# Photocatalytic CO_2_–to–Ethylene Conversion over Bi_2_S_3_/CdS Heterostructures Constructed via Facile Cation Exchange

**DOI:** 10.34133/2022/9805879

**Published:** 2022-10-19

**Authors:** Hai−Bo Huang, Ning Zhang, Jian−Ying Xu, Yu−Hang Xu, Ya−Feng Li, Jian Lü, Rong Cao

**Affiliations:** ^1^ Fujian Provincial Key Laboratory of Soil Environmental Health and Regulation, College of Resources and Environment, Fujian Agriculture and Forestry University, Fuzhou, China; ^2^ State Key Laboratory of Structural Chemistry, Fujian Institute of Research on the Structure of Matter, Chinese Academy of Sciences, Fuzhou, China; ^3^ School of Environmental Science and Engineering, Qingdao University, Qingdao, China; ^4^ State Key Laboratory of Photocatalysis on Energy and Environment, Fuzhou University, Fuzhou, China

## Abstract

Solar-driven CO_2_ conversion to multicarbon (C_2+_) products has emerged as a key challenge, yet this calls for a systematic investigation on the overall reaction process and mechanism at an atomic level based on the rational design of highly selective photocatalysts. Herein, we report the synthesis of compact Bi_2_S_3_/CdS heterostructures via facile cation exchange, by which a unique pathway of CO_2_–to–C_2_H_4_ photoconversion is achieved. Specifically, the BCS–30 shows an optimal C_2_H_4_ production rate of 3.49 *μ*mol h^−1^ g^−1^ based on the regulation of band structures and energy levels of photocatalysts by controlled growth of Bi_2_S_3_ at CdS surface. Both experimental and theoretical results (DFT calculations) identify Bi atoms as new catalytic sites for the adsorption of CO^*^ and formation of ^*^CO−^*^CO dimers that further hydrogenate to produce ethylene. Overall, this work demonstrates vast potentials of delicately designed heterostructures for CO_2_ conversion towards C_2+_ products under mild photocatalytic conditions.

## 1. Introduction

The conversion of carbon dioxide (CO_2_) into transportable chemicals has captured unparallel research attention because of the increasing quest for mitigating anthropogenic carbon emissions related to the consumption and depletion of traditional fuels such as coal, crude oil, and natural gases [1–4]. Moreover, the synthesis of multicarbon (C_2+_) products from CO_2_ is highly attractive due to their versatile applications in the chemical and energy industries, albeit the uncontrollable C–C coupling in thermocatalytic hydrogenation of CO_2_ over heterogeneous catalysts [5–7]. Nevertheless, this is extremely challenging due to the chemical inertness of CO_2_, the competing formation of methane (CH_4_), and the slow kinetics of proton and electron transport [8–10]. In particular, the high-energy density ethylene (C_2_H_4_) is one of the most important feedstocks with high commercial value for polymer production [11–13]. As known, a twelve-electron transfer process and multiple hydrogenation steps are involved in the conversion of CO_2_ to C_2_H_4_, which generally resulted in restricted conversion efficiency and unexpected products, i.e., CH_4_, carbon monoxide (CO) and formic acid (HCOOH). To address these issues, copper-based materials are currently the most promising catalysts for CO_2_ reduction into hydrocarbons via electrochemical catalysis based on alkaline flow cells [14–16], coupled to traditional iron-based catalysts and composite oxide catalysts (i.e., Cu−ZnO−Al_2_O_3_/zeolite) [17] that normally require additional input of power energy.

Most notably, great importance has been contemporarily focused on solar-driven photocatalysis that mimics the natural photosynthesis as an essential pathway to convert solar energy into clean and renewable chemical energy [18–20]. In a typical photocatalytic process, photogenerated charge carriers (electron and hole pairs) are induced upon the absorption of photons with energy greater than the bandgaps of semiconductor photocatalysts. Once photocarriers are separated and transported to the photocatalyst surface, redox reactions might take place by involving either donors (with holes) or acceptors (with electrons), thereby leading to the transformation of various substrates. Therefore, the discovery of viable and efficient photocatalysts is generally considered as the key engine for the development of environmental and energy photocatalysis including nitrogen (N_2_) fixation, hydrogen (H_2_) production, and CO_2_ reduction. In contrast to the production of C_2_H_4_ and other C_2+_ products via electrochemical CO_2_ reduction [21–24], however, not much success has been accomplished in CO_2_ reduction through viable photocatalytic systems hitherto.

One key step to produce C_2+_ products is the ^*^CO−^*^CO coupling that requires at least two catalytically active sites with an appropriate separation. Alternatively, a two-step tandem process is involving individually the conversion of CO_2_ to carbon monoxide (CO), followed by combination with H^+^ to form C_2+_ hydrocarbons, a process that requires the inhibition of methane (CH_4_) production. This could be achieved based on the rational design of functionally well-defined photocatalysts via sophisticated control of surface/interface structures for multiple-step chemical conversions [25]. To this end, heterostructure photocatalysts are promising in creating such functional surface/interface to realize tandem photocatalysis, since crystallographically matched phases within a heterostructure ideally satisfy the spatial control of catalytic sites in this scenario [26–28]. *In situ* growth of heterostructures can generate high-quality compact interface that facilitates efficient carrier transport. Furthermore, heterostructures combining narrow- and wide-bandgap semiconductors as visible-light-driven photocatalysts generally possess highly active sites and advanced structural features [29]. In this context, the cadmium sulfide (CdS) has a suitable bandgap (2.4 eV) that endows an excellent visible light absorption capacity for solar utilization [30], and thus CdS-based heterojunction materials formed by a simple synthetic method of cation-exchange exhibit excellent photocatalytic performance [31, 32]. Meanwhile, the bismuth sulfide (Bi_2_S_3_) with direct and narrow bandgap is considered as an excellent candidate to combine with CdS for CO_2_ photoreduction into value-added chemicals [33]. More importantly, the conduction band potential of Bi_2_S_3_ is more negative than most semiconductors, which allows suitable alignment of energy levels (bandgap, valence, and conduction band potentials) for CO_2_ photoreduction. However, it remains a daunting challenge for the controlled synthesis of viable heterostructure photocatalysts with the purpose of CO_2_ photoreduction into C_2+_ products.

We demonstrate herein the rational design and synthesis of heterostructure photocatalysts for tandem CO_2_–to–C_2_H_4_ conversion that couples individual steps within a chemically complicated pathway. The interface engineering via controlled cation exchange at the surface of CdS nanorods (NRs) gave rise to a series of Bi_2_S_3_/CdS (BCS–t) heterostructures that exhibited particularly efficient capacity for CO_2_ photoreduction under visible irradiation. Remarkably, as-prepared BCS–t photocatalysts achieved a unique CO_2_ reduction pathway to produce C_2_H_4_, of which the BCS–30 showed an optimal C_2_H_4_ production rate of 3.49 *μ*mol h^−1^ g^−1^. Overall, the current research outlines a new strategy for CO_2_ photoreduction into C_2+_ products by first time using a bismuth-based material.

## 2. Results and Discussion

**Figure 1:**
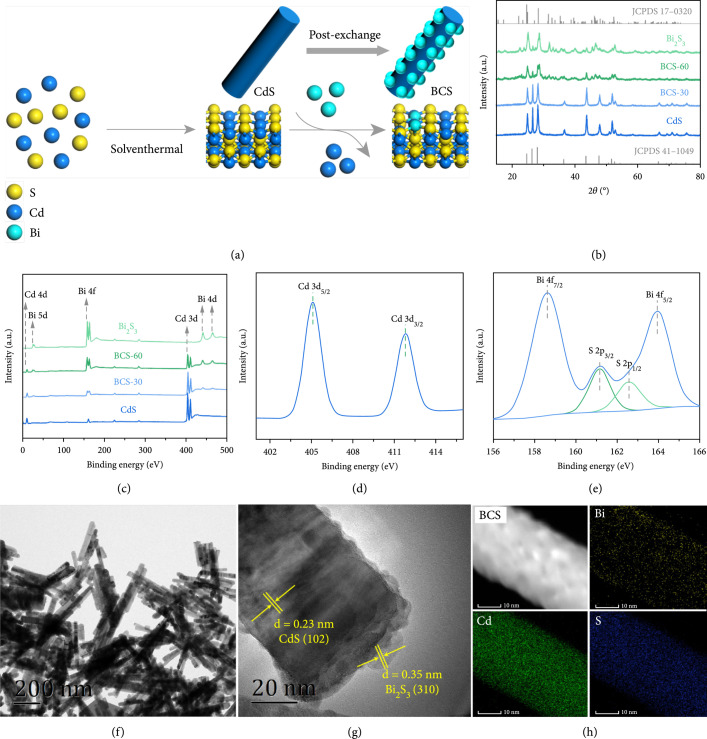
Characterizations of BCS–t samples. (a) Schematic illustration of the synthetic process of BCS–t composite materials, (b) PXRD patterns, and (c) XPS full spectra of CdS and BCS–t; XPS spectra of (d) Bi 4f and S2p, (e) Cd 3d, (f) TEM, (g) HR–TEM, and (h) elemental mapping analyses of BCS–30.

We successfully synthesized BCS–t composites with the design strategy illustrated in Figure 1(a). Phase and composition of CdS, Bi_2_S_3_, and BCS–t heterostructures were studied by powder X–ray diffractions (PXRD) and X–ray photoelectron spectrometry (XPS). As shown in Figure 1(b) and S1, the main characteristics of pristine CdS were assigned to the hexagonal phase of wurtzite CdS (JCPDS: 41–1049) [34]. Upon cation exchange with Bi^3+^, diffraction peaks of the orthogonal Bi_2_S_3_ (JCPDS: 17–0320) were observed, which was rational since the Bi_2_S_3_ possesses a smaller $K_{\text{sp}}$ (ca. $1.0\times 10^{-97}$) than CdS (ca. $8.0\times 10^{-27}$), and thus, CdS could partially transform to Bi_2_S_3_ in the presence of Bi^3+^ ions [35]. Moreover, the intensity of hexagonal CdS characteristics gradually decreased, while those of orthorhombic Bi_2_S_3_ peaks evidently increased with extended time of ion exchange. A nearly complete exchange was achieved after 120 min, which resulted in the formation of an orthorhombic Bi_2_S_3_ phase [35]. In addition, XPS spectra was performed to analyze the surface chemical composition and valence state of various elements in these samples. The XPS survey spectrum clearly showed the existence of Cd, S, and Bi elements in the representative BCS–30 (Figure 1(c)). As shown in Figure 1(d), the two peaks at binding energies of ca. 158.6 eV and ca. 163.9 eV corresponded to Bi 4f_5/2_ and Bi 4f_7/2_ orbits, respectively, indicating the presence of Bi^3+^ in BCS–30 [36]; the characteristics at ca. 161.6 eV and ca. 162.8 eV were assigned to S 2p_3/2_ and S 2p_1/2_, respectively, and the split energy of ca.1.2 eV was the fingerprint for S^2–^ [37]; the two peaks around ca. 404.8 eV and ca. 411.5 eV were attributable to Cd 3d_5/2_ and Cd 3d_3/2_, respectively, which were typical for Cd^2+^ in sulfides (Figure 1(e)) [38]. Consequently, the elemental composition of all samples was analyzed by means of the inductive coupled plasma emission spectrometer (ICP) as presented in Supplementary Table 1.

To gain further insights into structural details of BCS–t heterostructures, microstructure and morphology were investigated by SEM, TEM, and HR–TEM (Figures 1(f) and 1(g), and Figures S2–S4). The pristine CdS showed a rod-like morphology with an average diameter of ca. 50 nm and length of 200 nm to 300 nm. Morphology of the representative BCS–30 revealed that Bi_2_S_3_ nanoparticles were decorated on the surface of CdS nanorods (NRs) with high dispersity, and no clear change in the microstructure of CdS NRs was observed. In the HRTEM image (Figure 1(g)), lattice fringes with spacing of ca. 0.23 nm and ca. 0.35 nm were ascribed to the (1 0 2) crystal plane of hexagonal CdS and (3 1 0) crystal plane of orthorhombic Bi_2_S_3_, respectively. Moreover, elemental mapping analyses of BCS–30 were studied to verify the successful and homogeneous dispersion of Bi_2_S_3_ on CdS NRs. As shown in Figure 1(h), the mapping images of Bi, Cd, and S contents suggested a uniform distribution of each element in BCS–t samples. It was therefore concluded that Bi_2_S_3_ nanoparticles was successfully deposited on the surface of CdS NRs via cation exchange, which might create intimate contacts and compact interface between Bi_2_S_3_ and CdS counterparts.

UV–vis diffuse reflectance spectra (DRS) of CdS and Bi_2_S_3_ are displayed in Figure S5. The pristine CdS showed an absorption edge around 525 nm, and Bi_2_S_3_ showed strong absorption in the whole ultraviolet–visible region. In comparison, the light absorption range of BCS–t heterostructures was further extended from UV to the visible light region. Therefore, the corresponding bandgaps of CdS and Bi_2_S_3_ were estimated to ca. be 2.36 and ca. 2.04 eV (Figures S4b and S4d), respectively. To better understand the effect of band structure on charge separation efficiency and photocatalytic activity, Mott−Schottky diagrams were investigated. As shown in Figure S6, the positive tangent slopes in the Mott–Schottky indicated that both CdS and Bi_2_S_3_ were typical n-type semiconductors [39, 40]. Thus, the flat band potential ($E_{\text{FB}}$) of CdS and Bi_2_S_3_ were calculated as ca. −1.25 and ca. −0.77 V vs. Ag/AgCl (ca. −1.05 and ca. −0.57 V vs. NHE), respectively. Furthermore, the valence band (VB) of CdS and Bi_2_S_3_ were determined to be ca. 1.31 and ca. 1.47 V vs. NHE, respectively. Based on the energy alignment, Bi_2_S_3_ and CdS in BCS–t can be organized into either Z-scheme or type-II heterojunction.

**Figure 2:**
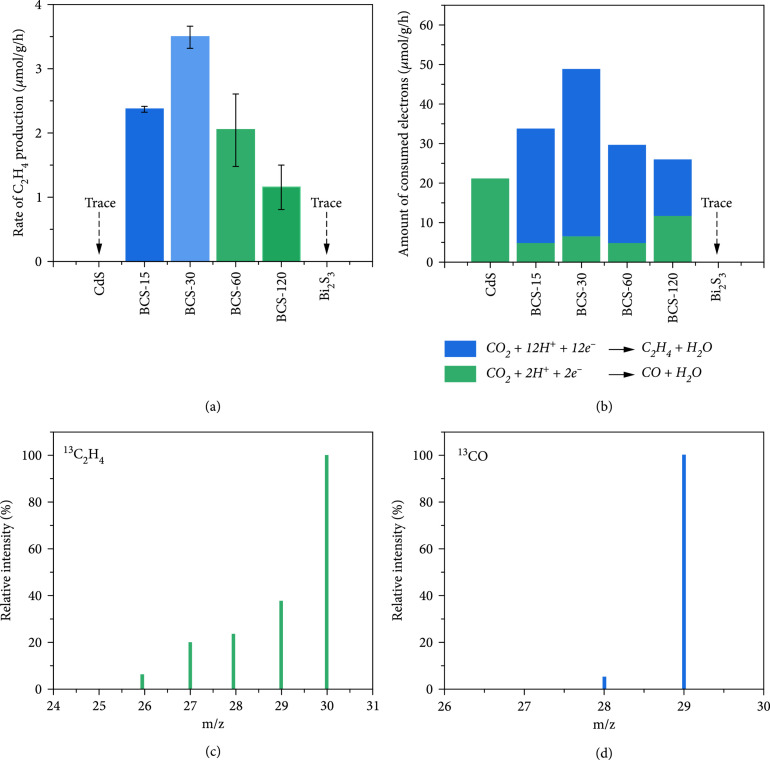
Photocatalytic performances. (a) Yields of C_2_H_4_, (b) charge transfer in CO_2_ photoreduction over CdS, Bi_2_S_3_, and BCS–t under visible irradiation, and (c) mass spectra of ^13^C_2_H_4_ (m/z=30) and (d) ^13^CO (m/z=29) generated under ^13^CO_2_ atmosphere.

In the system of CO_2_ photoreduction with various catalysts, CO and C_2_H_4_ were identified as major products of CO_2_ photoreduction, and H_2_ was produced via the reduction of water vapor. As shown in Figure S7a, the CO production rate of pristine CdS was ca. 10.5 *μ*mol g^−1^ h^−1^ which was considerably higher than those of BCS–t heterostructures. Notably, CO was the sole detectable product of CO_2_ photoreduction with CdS NPs, whereas C_2_H_4_ production was observed for BCS–t photocatalysts. Since the single Bi_2_S_3_ did not produce CO products, the active site of CO production was likely at the surface of CdS, and thus, a Z-scheme heterojunction might form between CdS and Bi_2_S_3_ counterparts. Moreover, the C_2_H_4_ production rate of BCS–t was synergically regulated by Bi_2_S_3_ : CdS ratios as neither of the individual CdS nor Bi_2_S_3_ was able to yield C_2+_ products. Especially, the BCS–30 showed the maximum yield of 3.49 *μ*mol h^−1^ g^−1^ under visible irradiation, which was roughly 1.5, 1.7, and 3.0 times those of BCS–15, BCS–60, and BCS–120, respectively (Figure 2(a)). Therefore, it was clear that the C_2_H_4_ production capacity of heterostructure photocatalysts was readily tuned via delicate control on the process of cation exchange, which demonstrated that moderate decoration of Bi_2_S_3_ at CdS surface was crucial to modulate photocatalytic activity of BCS–t.

On the other hand, the H_2_ production of BCS–t followed a similar trend to that of C_2_H_4_ production, in which the BCS–30 exhibited the highest production rate of 2971 *μ*mol h^−1^ g^−1^ (Figure S7b). Moreover, the amount of consumed electrons in CO_2_ photoreduction over BCS–t heterostructures was calculated based on the production of CO (2 e^–^) and C_2_H_4_ (12 e^–^). Results showed that BCS–t photocatalysts generally processed enhanced electron utilization than those of CdS and Bi_2_S_3_, among which BCS–30 achieved the highest amount of electron transport for CO_2_ photoreduction (Figure 2(b)). Moreover, BCS−30 exhibited the best photoelectron selectivity for CO_2_ photoreduction to carbonaceous products (Figure S7c). In addition, the applicability of BCS–30 was tested by long-term experiments, in which the photocatalyst exhibited both stable photocatalytic activity (Figures S8a–S8c) and phase crystallinity (Figure S8d), which was also demonstrated by TEM (Figure S9) and XPS (Figure S10) recorded on recycled BCS–30.

In order to trace the origin of carbonaceous products, ^13^CO_2_ isotope labeling experiments were performed. As shown in Figures 2(c) and 2(d), gas chromatography and mass spectrometry (GC–MS) analysis clearly identified ^13^C_2_H_4_ and ^13^CO, which indicated the input of CO_2_ as viable carbon sources. Moreover, control substrate experiments under the same catalytic conditions without adding H_2_O and MeCN or by replacing CO_2_ with Ar could give rise to only trace or extremely low amount of carbon products (Figures S11a–S11c). Alternatively, the BCS–30 remained producing a detectable amount of ethylene with inorganic Na_2_S and Na_2_SO_3_ aqueous solution as sacrificial reagents. Surprisingly, significant amount of C_2_H_4_ were produced by replacing the CO_2_ with CO as the reaction substrates (Figure S8d). These are conclusive evidence for viable C_2_H_4_ production, and the formation of CO might be a key step that initiates CO_2_–to–C_2_H_4_ conversion with BCS–t photocatalysts.

The photoluminescence (PL) quenching technique was used to study the separation efficiency of photoinduced electron–hole pairs in CdS, Bi_2_S_3_, and BCS–t. As shown in Figure S12a, the CdS exhibited strong characteristic PL emission centered at ca. 750 nm, whereas BCS–t displayed largely decreased PL intensity with increasing Bi_2_S_3_ contents. PL results demonstrated that the separation efficiency of photogenerated electrons and holes in BCS–t heterostructures was markedly improved by virtue of the synergistic effect between CdS and Bi_2_S_3_ counterparts. The improved separation efficiency of photocarriers in these heterostructure photocatalysts was further confirmed by electrochemical impedance spectroscopy (EIS) analyses (Figure S12b), in which the BCS–t generally showed smaller arc resistance radius than the pristine CdS, suggesting much enhanced separation and transfer of charge carriers in BCS–t heterostructures.

**Figure 3:**
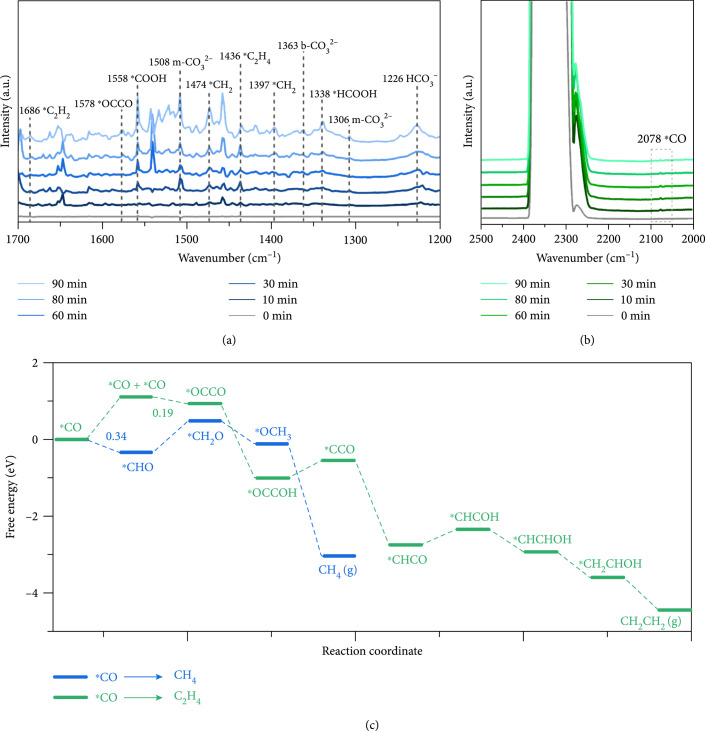
Mechanism of CO_2_ photoconversion pathways. (a, b) In situ DRIFTS for BCS–30 in the atmosphere of CO_2_ under visible light irradiation and (c) the free energy diagram of photoreduction CO_2_ to CH_4_ and C_2_H_4_ on BCS–30 at U=0V.

*In situ* diffuse reflectance infrared Fourier transform spectroscopy (DRIFTS) was performed to monitor the possible intermediate species during the process of CO_2_ photoreduction in this current system. First, CO_2_ and H_2_O molecules were absorbed at the surface of BCS–t for 10 min in dark. Upon visible light irradiation, new peaks that gradually intensified with reaction progressing were observed in DRIFTS spectra (Figures 3(a) and 3(b)). These peaks were assigned to HCO_3_^−^ (1226 cm^–1^), m–CO_3_^2–^ (1306/1508 cm^–1^), b–CO_3_^2–^ (1363 cm^–1^), ^*^HCOOH (1338 cm^–1^), and ^*^COOH (1558 cm^–1^). More importantly, absorption characteristics of key intermediates, including ^*^OCCO (1578 cm^–1^), ^*^CH_2_ (1397/1474 cm^–1^), and ^*^C_2_H_4_ (1436/1686 cm^–1^), were successfully captured as a proof of photocatalytic CO_2_–to–C_2_H_4_ conversion [41]. However, similar tests with the CdS photocatalyst solely revealed absorption peaks of ^*^COOH and ^*^CO, which supported the unique role of BCS–t photocatalysts in the formation of key intermediates for CO_2_–to–C_2_H_4_ production conversion (Figure S13).

**Figure 4:**
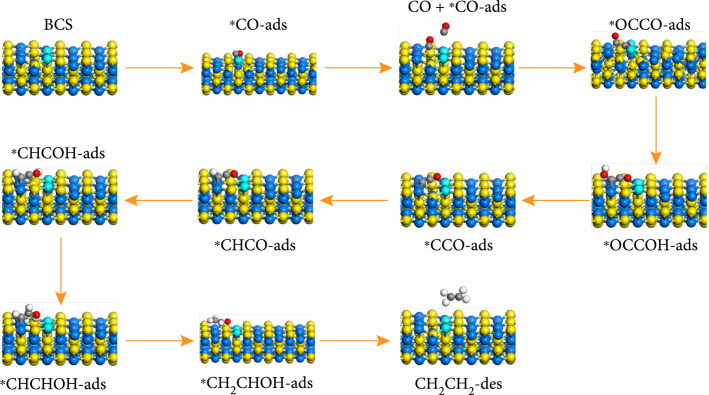
Possible intermediates at the surface of BCS–30 via the C_2_H_4_ pathway.

To further reveal the mechanism of CO_2_–to–C_2_H_4_ conversion in this current system, DFT calculations were performed to investigate the pathway of CO_2_ photoreduction, as detailed in the Supporting Information. First, the conversion products CH_4_ and C_2_H_4_ are a competing reaction; however, the rate determining step (RDS) for both pathways is that the coupling of ^*^CO to form ^*^OCCO has a lower corresponding free energy than the hydrogenation of ^*^CO to form ^*^CHO (Figure 3(c)). This means that the final product of the ground will not be methane, but will go to the option of producing ethylene. Computational intermediate models formed by CH_4_ and C_2_H_4_ are provided in the Supporting Information (Figure 4 and Figure S14). Then, we performed calculations for the various pathways from CO_2_ to ethylene, and the most likely route is pathway4 due to the lowest RDS (Figures S15 and S16). The production of C_2_H_4_ from ^*^CO_2_^–^ possibly occurs through ^*^CO_2_^–^ → ^*^CO → ^*^OCCO → ^*^CHCO → ^*^CHCOH → ^*^CHCHOH → ^*^CH_2_CHOH → ^*^C_2_H_4_ chain reactions, in which Bi atoms are new catalytic sites for the adsorption of CO^*^ and the formation of ^*^CO−^*^CO dimers, which further hydrogenate to produce ethylene.

Based on the above characterizations, a mechanism of solar-driven CO_2_–to–C_2_H_4_ conversion in the BCS–t is proposed as follows. CO_2_ and H_2_O molecules are first adsorbed at the surface of photocatalyst, which is excited by visible irradiation to generate photocarriers (h^+^ and e^–^). Meanwhile, electrons quickly transport to catalyst surface and participate in CO_2_ photoreduction, and then, photogenerated electrons are accumulated at CBs of CdS and Bi_2_S_3_ in a BCS–t heterostructure, in which electrons also transfer from the CB of Bi_2_S_3_ to the VB of CdS through a Z-scheme electron migration scheme. In such a way, holes are accumulated at a lower energy level, and the redox ability is much stronger to achieve CO_2_ reduction while preventing the photocorrosion of CdS. CO_2_ molecules are readily reduced by photoelectrons to yield COOH, which is then hydrogenated to produce CO. Further hydrogenation via CO leads to dimerization into C_2_H_x_O [42]. Finally, these intermediates are further reduced to C_2_H_4_.

## 3. Conclusion

Although it appears extremely challenging to produce value-added C_2+_ chemicals from CO_2_ photoreduction, we present here a facile cation exchange strategy to synthesize Bi_2_S_3_/CdS (BCS–t) heterostructures which show vast potentials for solar-driven CO_2_–to–C_2_H_4_ conversion. The proposed synthetic pathway successfully creates catalytically active and compact interface between Bi_2_S_3_ and CdS counterparts in BCS–t, which leads to tandem photocatalysis for C_2_H_4_ production. Upon controlled growth of Bi_2_S_3_ at the surface of CdS, BCS–30 shows a maximal C_2_H_4_ production rate due to an optimal ratio of the two components. This work thus provides new insights into the design of viable photocatalysts for solar-driven CO_2_ photoreduction towards C_2_H_4_ production.

## 4. Materials and Methods

### 4.1. Preparation of CdS Nanorods (CdS NRs)

All chemicals were of analytical grade and directly used without further purification. The CdS nanorods were synthesized in a solvothermal system using ethylenediamine as both the structure directing agent and solvent. First, 3.0 mmol Cd(NO_3_)_2_·4H_2_O and 9.0 mmol CS(NH_2_)_2_ were dissolved in 30 mL mixed solvents of ethylenediamine and water (v:v=2:1) for 30 min with vigorous stirring. Then, the above solution was transferred to a 50 mL solvothermal autoclave, and the reaction was kept at 180°C for 12 h, followed by cooling naturally to room temperature. The orange−yellow product of CdS NRs was collected by centrifugation and washed several times with deionized water and dried at 70°C overnight.

### 4.2. Preparation of Bi_2_S_3_/CdS (BCS) Heterostructures

Bi_2_S_3_/CdS heterostructures were prepared by a simple ion exchange method in ethylene glycol (EG) solution. Briefly, 0.5 mmol CdS NRs were dissolved in 20 mL EG and sonicated for 15 min, followed by the addition of 1.5 mmol Bi(NO_3_)_2_·5H_2_O was added under constant stirring at 75°C for different time (15/30/60/120/240 min). The resultant brownish−yellow solid precipitates were collected by centrifugation and washed several times with deionized water and then were dried at 70°C overnight. The as-prepared BCS samples were denoted as BCS–t (t=15, 30, 60, and 120). Complete exchange of Cd^2+^ with Bi^3+^ was realized after 240 min, and the sample was therefore denoted as Bi_2_S_3_.

### 4.3. Photocatalytic CO _2_ Reduction

The photocatalytic CO_2_ reduction was performed by using a sealed quartz reactor with an optically transparent quartz cover. In a typical experiment, 10 mg photocatalyst was loaded into a 210 mL quartz glass reactor containing 6.0 mL acetonitrile, 2.0 mL deionized water, and 2.0 mL triethanolamine. Before light irradiation, the reaction system was degassed with vacuum, and then photocatalytic reactions were carried out using a 300 W Xe–arc lamp (PLS–SX300D, Beijing) equipped with a UV cut filter (wavelength 400 nm) as the light source. Subsequently, the reaction system was vacuum degassed and backfilled with high-purity CO_2_ gas (99.99%), which was repeated for three times to ensure the reactor was finally backfilled with pure CO_2_. Before photocatalysis, dark reaction was first applied for 1 h to ensure the adsorption equilibrium. During a photocatalytic reaction, 1.0 mL gas was extracted hourly from a glass reactor using gas chromatography (GC–7820A, Shimadzu) equipped with flame ionization detector (FID) and capillary column (GC–GASPRO) and used for subsequent gas content/concentration analyses. The stability of photocatalysts was evaluated by continuous reactions using recovered solids and fresh solutions. Control substrate experiments were tested under the same catalytic condition without the addition of H_2_O or MeCN or by replacing CO_2_ with Ar. Alternatively, the photocatalytic reaction is carried out by using Na_2_S and Na_2_SO_3_ (NNS) aqueous solution as sacrificial agents. In the isotope tracking experiments by substituting ^13^CO_2_ for ^12^CO_2_ gas to demonstrate the origin of the product, other experimental conditions are the same as the photocatalytic CO_2_ reduction.

### 4.4. In Situ Diffuse Reflectance Infrared Fourier Transform Spectroscopy (DRIFTS)

Thermo Scientific Nicolet 6700 spectrometer was used for the in situ DRIFTS over as–samples. An average of 128 scans was taken for each spectrum, which had a resolution of 4 cm^–1^. CO_2_ flow (40 mL/min) was bubbled into MeCN/H _2_O/TEOA (3 : 1 : 1) and then passed through the sample cell. The visible light irradiation was shed on the powder through a quartz window of the sample cell. The CO_2_ gaseous was kept steady before the light irradiation.

### 4.5. Computational Details

All the density functional theory (DFT) calculations were performed using the Vienna ab initio simulation package (VASP) [43–46]. The projector augmented wave (PAW) method was used to describe the interactions between ion cores and valence electrons [47]. The GGA–PBE was employed to describe the exchange–correlation functional [48, 49]. The Kohn–Sham equations were expanded in a plane wave basis set with a cutoff energy of 400 eV. The Brillouin zone sampling was performed using 3×3×1 Monkhorst–Pack k-point grid [50]. According to XPS, the (102) surface of CdS was chosen to investigate the activity of Bi doped Bi@CdS catalyst. For the investigated catalyst, a 4×4 supercell model with three atomic layers were constructed. During the optimization, the most bottom layer is fixed, and the other two layers are relaxed.

## Data Availability

Data supporting the findings of this study are available in the main text or the supporting materials.

## Supplementary Materials

Supplementary Materials Table S1: elemental analysis of BCS–t heterostructures. Figure S1: PXRD patterns of the CdS and BCS–t. Figure S2: SEM images of (a) CdS and (b) BCS–30. Figure S3: (a) TEM and (b) HR–TEM images of the CdS. Figure S4: (a) and (b) TEM, (c) HR–TEM, and (d–h) elemental distribution images of the BCS–30. Figure S5: (a) UV–vis DRS and (b) K–M plots of CdS; (c) UV–vis DRS and (d) K–M plots of Bi_2_S_3_. Figure S6: Mott–Schottky plots of (a) CdS and (b) Bi_2_S_3_. Figure S7: yields of (a) H_2_ and (b) CO in CO_2_ photoreduction over CdS and BCS–t under visible irradiation. Figure S8: long-term experimental photocatalytic activity of (a) C_2_H_4_, (b) CO, (c) H_2_ of the BCS–30, and (d) PXRD patterns of the BCS–30 before and after the long-term photocatalytic experiment. Figure S9: (a) TEM, (b) HR–TEM, and (c) elemental distribution images of the BCS–30 before and after the long-term photocatalytic experiment. Figure S10: (a) XPS full spectra, XPS spectra of (b) Cd 3d and (c) Bi 4f and S2p of the BCS–30 before and after the long-term photocatalytic experiment. Figure S11: photocatalytic activity of (a) H_2_, (b) CO, (c) C_2_H_4_ ($\text{NNS}=\text{N}{\text{a}}_{2}\text{S}/\text{N}{\text{a}}_{2}\text{S}{\text{O}}_{3}$ aqueous solution) of the BCS–30, and (d) comparison of ethylene activity in different gas atmosphere (inset: H_2_ production rate). Figure S12: (a) room temperature PL and (b) electrochemical impedance spectra of CdS, Bi_2_S_3_, and BCS–t. Figure S13: *in situ* DRIFTS for CdS in CO_2_ atmosphere under visible light irradiation. Figure S14: structural evolution of intermediates in the CH_4_ pathway on the BCS surface. Figure S15: the free energy diagram of the various pathways of CO_2_–to–C_2_H_4_ photoreduction on the BCS at U=0V. Figure S16: the structures of intermediates in various C_2_H_4_ production pathways on the BCS surface. (a) The CH_2_CHO pathway and (b) the CHC pathway.
