# Supplementary material for: Photocatalytic CO2–to–Ethylene Conversion over Bi2S3/CdS Heterostructures Constructed via Facile Cation Exchange
Source: Research (Wash D C). 2022 Oct 19;2022:9805879. doi: 10.34133/2022/9805879 (PMC11030114; doi:10.34133/2022/9805879)

**Supporting Material**

**Photocatalytic CO_2_–to–ethylene conversion over Bi_2_S_3_/CdS heterostructures constructed via facile cation exchange**

Short title: **CO_2_–to–ethylene photoconversion**

Hai−Bo Huang^1,2,3^, Ning Zhang^1^, Jian−Ying Xu^1^, Yu−Hang Xu^1^, Ya−Feng Li^4^, Jian Lü^1,4*^, Rong Cao^2*^

^1^Fujian Provincial Key Laboratory of Soil Environmental Health and Regulation, College of Resources and Environment, Fujian Agriculture and Forestry University, Fuzhou China.

^2^State Key Laboratory of Structural Chemistry, Fujian Institute of Research on the Structure of Matter, Chinese Academy of Sciences, Fuzhou China.

^3^School of Environmental Science and Engineering, Qingdao University, Qingdao China.

^4^State Key Laboratory of Photocatalysis on Energy and Environment, Fuzhou University, Fuzhou China

*Correspondence should be addressed to Jian Lü: jian_lu_fafu@163.com and Rong Cao: rcao@fjirsm.ac.cn.

- 1. **Photoelectrochemical measurements**

In a standard three–electrode system, photoelectrochemical test was performed on an electrochemical analyzer (Zahner, Germany), with aqueous Na_2_SO_4_ solution (0.2 M, pH = 6.8) as the supporting electrolyte. The suspension was prepared by mixing 5.0 mg photocatalyst with 1.0 mL ethanol and 50 μL Nafion under sonication for 1 h. Indium tin oxide (ITO) glass (deposition area of 1.0 cm^2^) was used as the working electrode. Mott–Schottky plots of CdS and Bi_2_S_3_ were measured in 0.2 M Na_2_SO_4_ aqueous solution at frequencies of 500, 1000 and 1500 Hz, respectively, and calculated using the following equation, where C is the space charge capacitance in the semiconductor; N_D_ is the carrier density; e is the elemental charge; ε_0_ is the permittivity of the vacuum; ε is the relative permittivity of the semiconductor; E is the applied potential; E_FB_ is the flat band potential; T is the temperature and K_B_ is the Boltzmann constant.
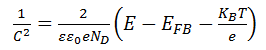


The bandgap of Bi_2_S and CdS was calculated by the following equation: *αhν* = A(h*ν*–E_g_)^1/2^, where *α* is the adsorption coefficient and *hν* is the photon energy; A is a constant; and E_g_ is the bandgap. Transient photocurrent was recorded under visible light irradiation. Electrochemical impedance spectroscopy (EIS) was collected at off–line potential in a frequency range of 100 to 0.01 Hz and a modulation amplitude of 5.0 mV.

- 1. **Characterizations**

Powder X–ray diffraction (PXRD) patterns were collected using a Rigaku Miniflex 600 X–ray diffractometer with Cu Kα radiation (λ = 0.154 nm). Transmission electron microscopy (TEM) and high–resolution TEM (HR–TEM) images were recorded by using an FEIT 20 working at 200 kV. The inter–planer distances and the inverse Fast Fourier Transform (FFT) were calculated using the Digital Micrograph software. X–ray photoelectron spectroscopy (XPS) measurements were performed on a Thermo Fisher ESCALAB 250Xi spectrometer with Al Kα X–ray source (15 kV, 10 mA). In order to compensate effects related to charge shifts C 1s peak at 284.6 eV was used as internal standard. Diffuse reflectance spectra (DRS) were recorded on a Shimadzu UV–vis spectrophotometer (UV–2550) with BaSO_4_ as the background. The photoluminescence (PL) spectra and time–resolved fluorescence emission spectrum were collected on a FLS 980 fluorometer spectrometer at room temperature.

**Table S1.** Elemental analysis of BCS–t heterostructures.

| **Sample** | **Cd%** | **Bi%** | **S%** | **Cd:Bi** |
| --- | --- | --- | --- | --- |
| BCS–15 | 76.1 | 8.8 | 14.2 | 16.2 : 1 |
| BCS–30 | 79.4 | 10.1 | 9.1 | 14.6 : 1 |
| BCS–60 | 69.7 | 21.8 | 7.5 | 6.0 : 1 |
| BCS–120 | 47.6 | 40.8 | 9.1 | 2.2 : 1 |

**Figure S1.** PXRD patterns of the CdS and BCS-t.


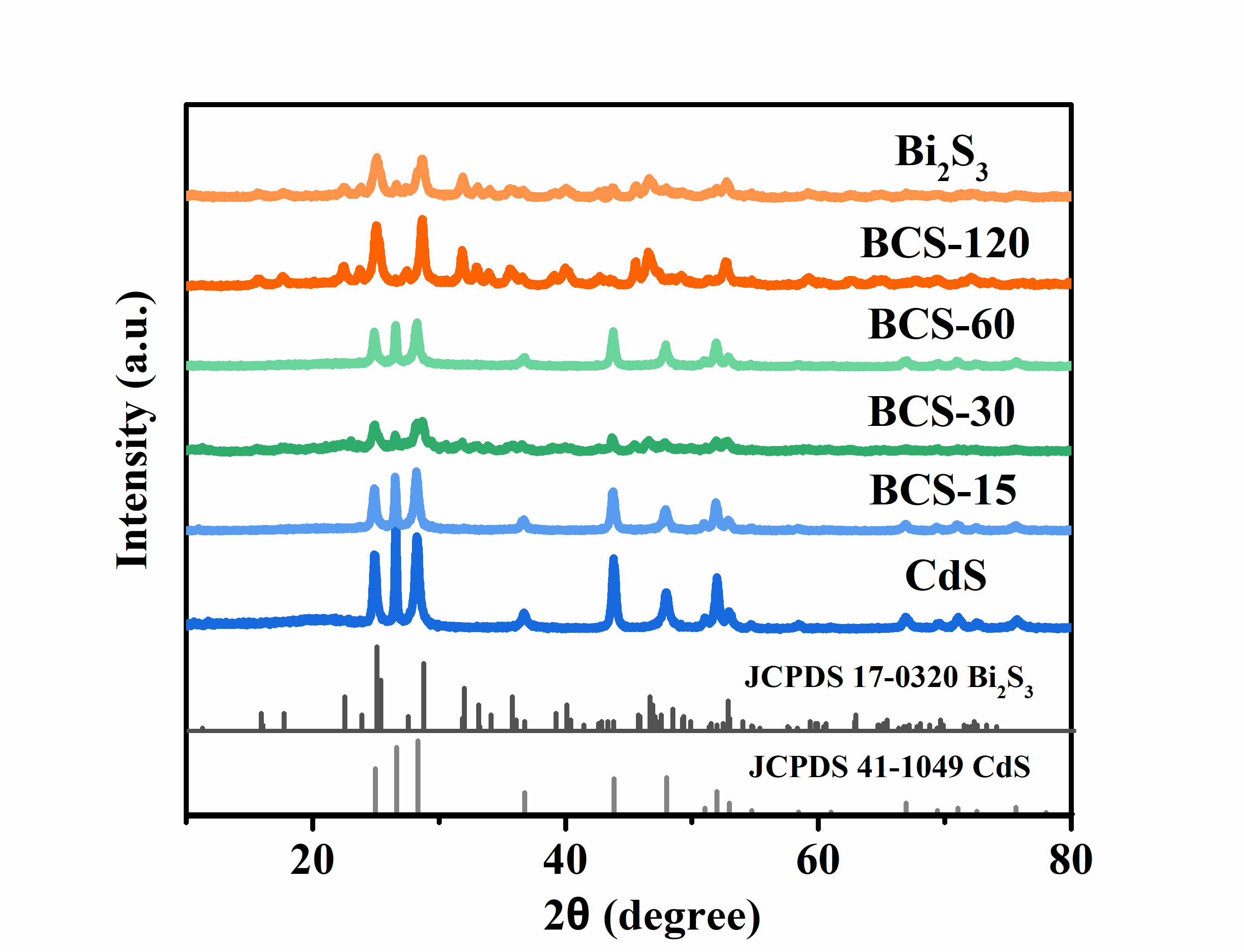


**Figure S2.** SEM images of (a) CdS and (b**)** BCS–30.


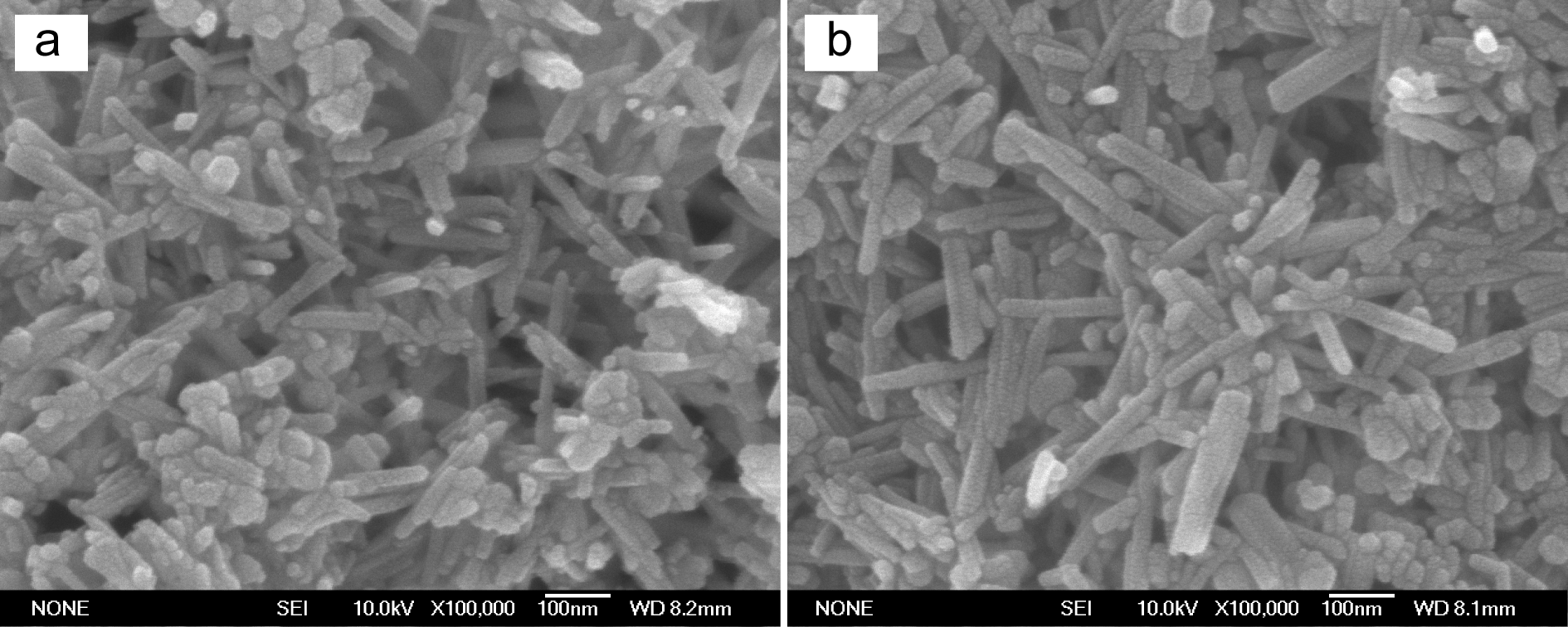


**Figure S3.** (a) TEM and (b) HR–TEM images of the CdS.


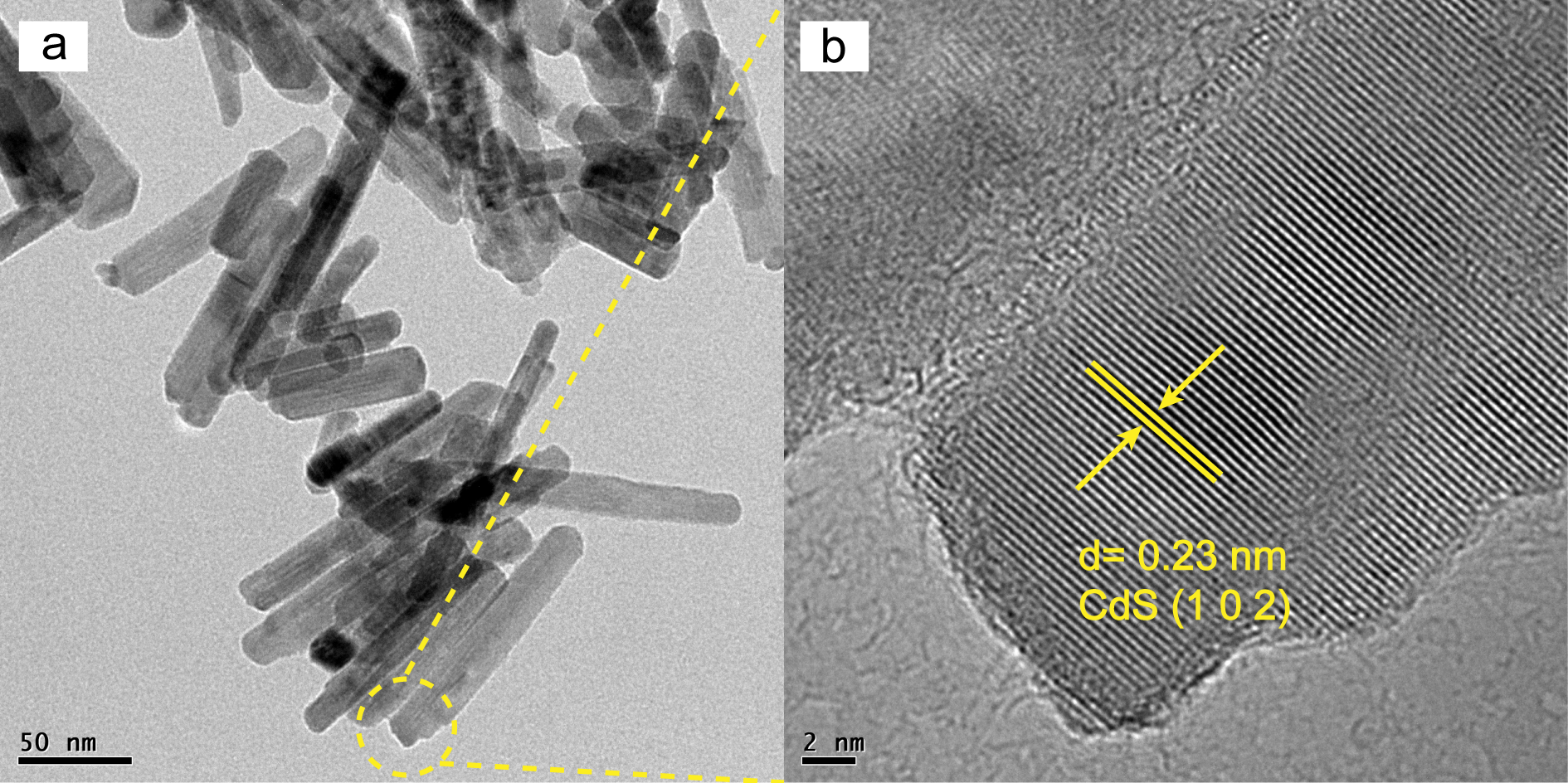


**Figure S4.** (a) and (b) TEM, (c) HR–TEM, and (d–h) elemental distribution images of the BCS–30.


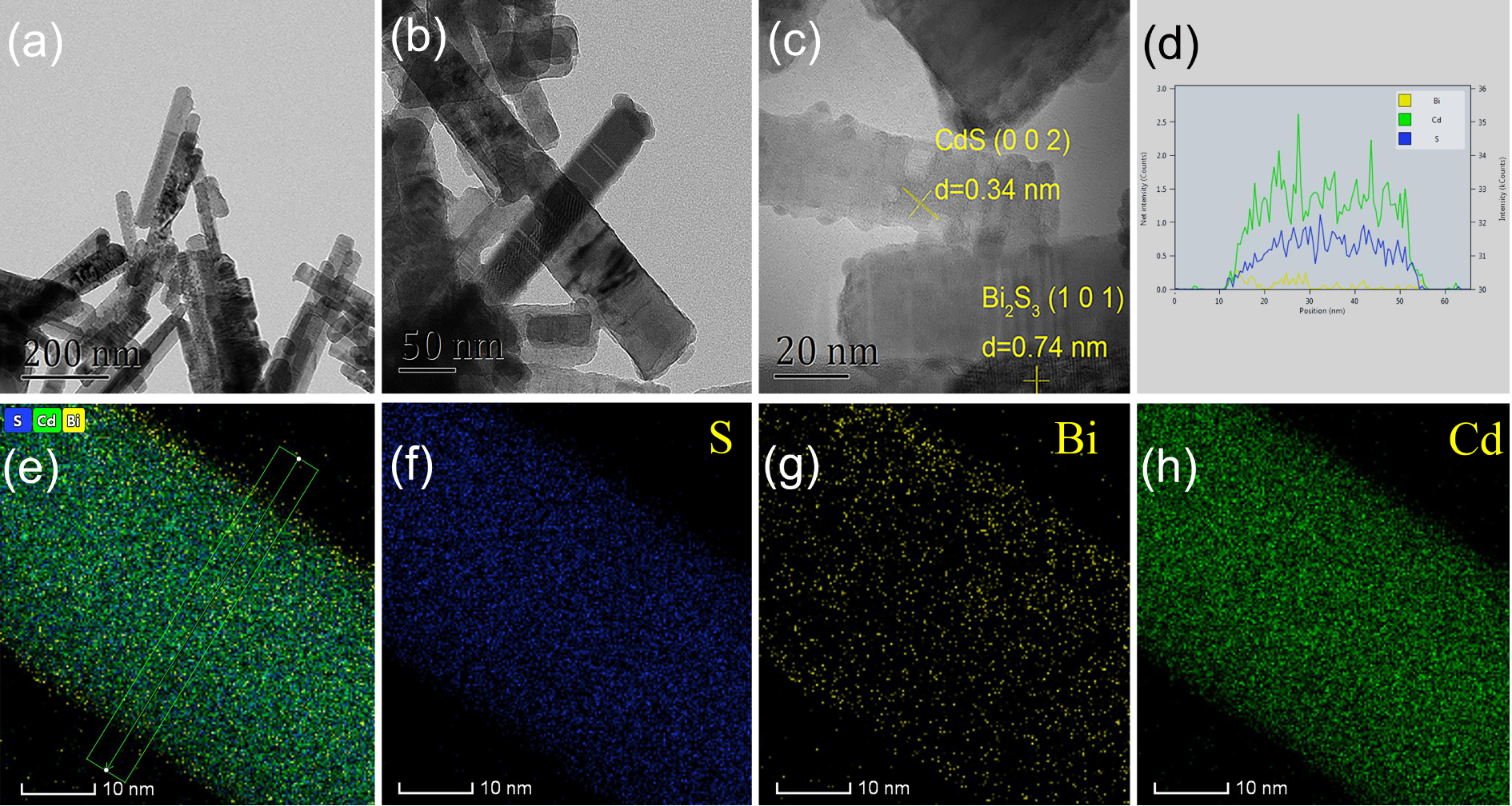


**Figure S5.** (a) UV–vis DRS and (b) K–M plots of CdS; (c) UV–vis DRS and (d) K–M plots of Bi_2_S_3_.

**
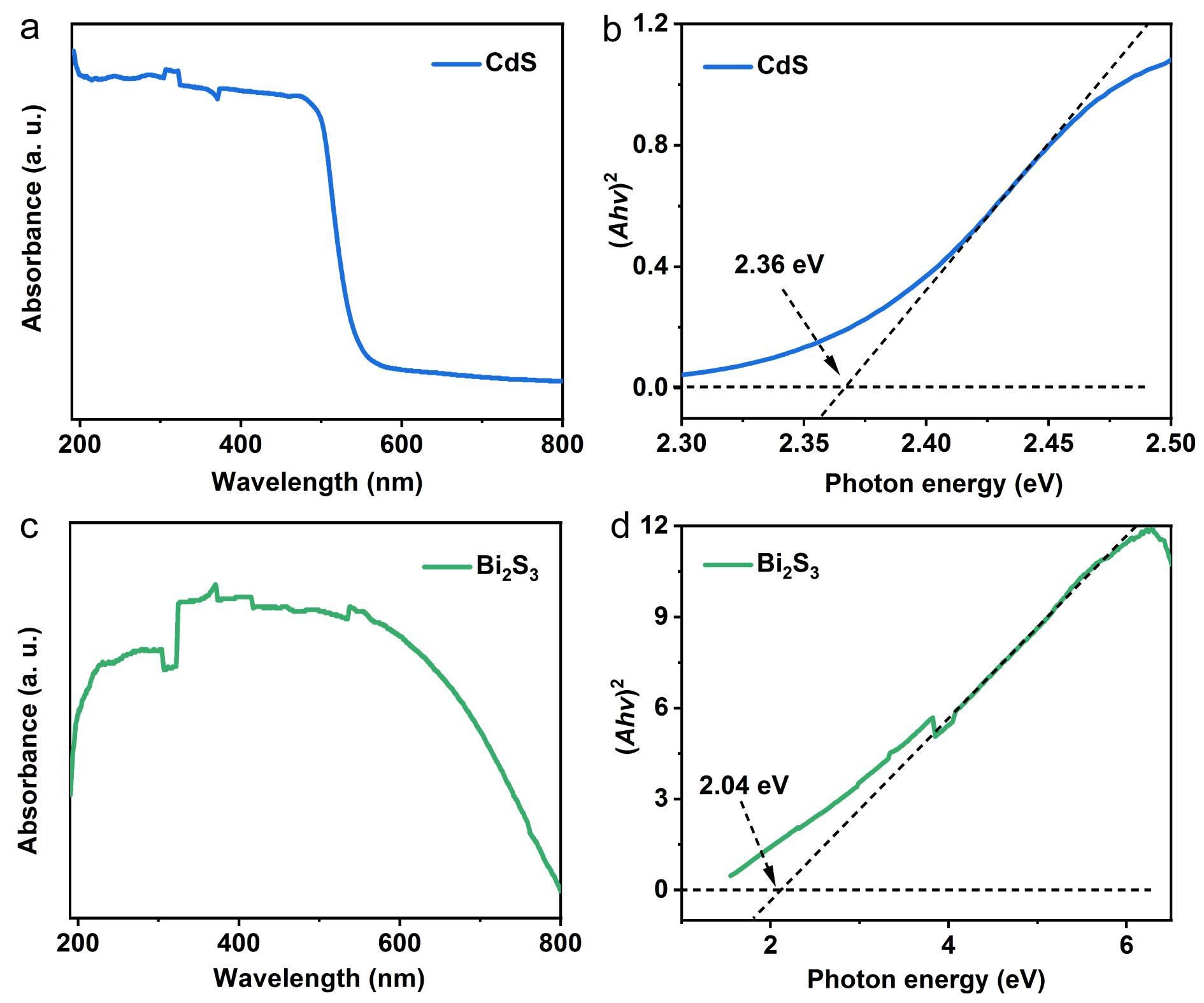
**

**Figure S6.** Mott–Schottky plots of (a) CdS and (b) Bi_2_S_3_.

**
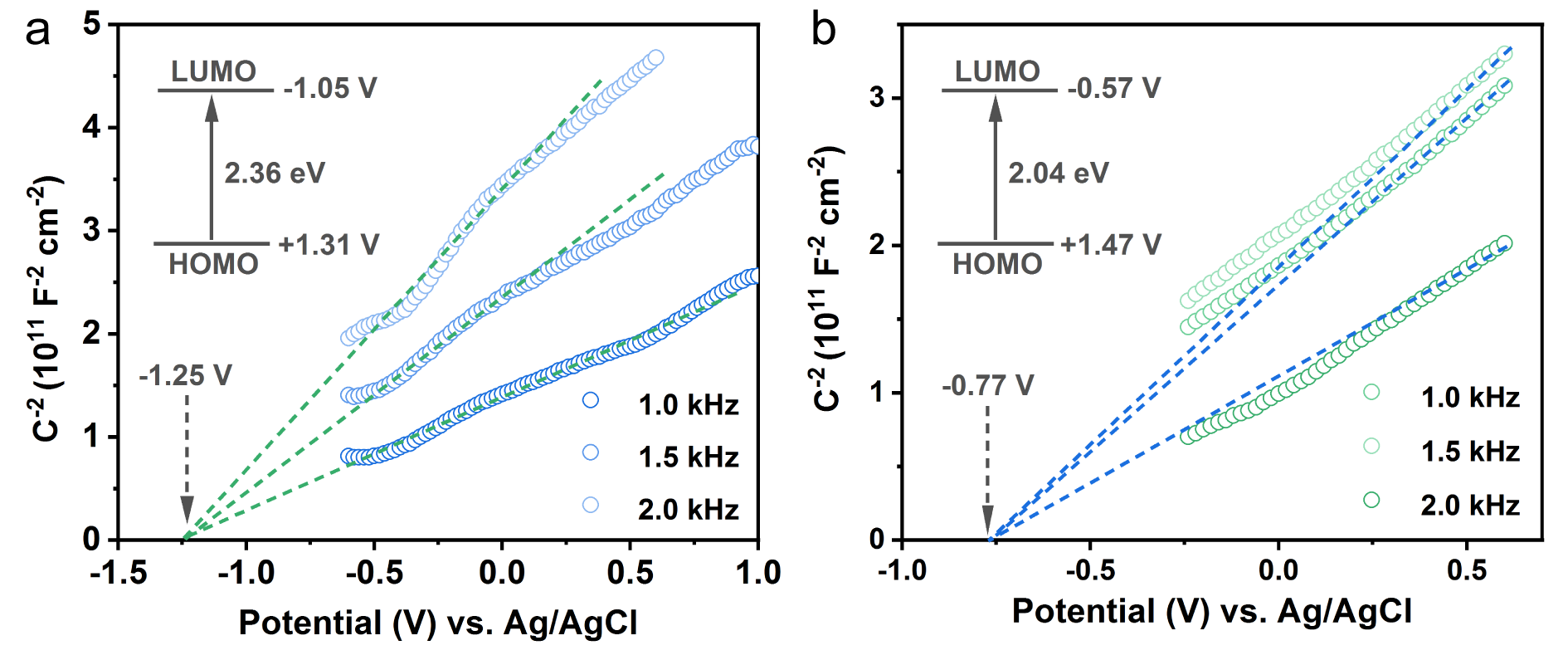
**

**Figure S7.** Yields of (a) H_2_ and (b) CO in CO_2_ photoreduction over CdS and BCS–t under visible irradiation; and (c) selectivity of photoelectrons for the photoreduction of CO_2_ to carbonaceous products.


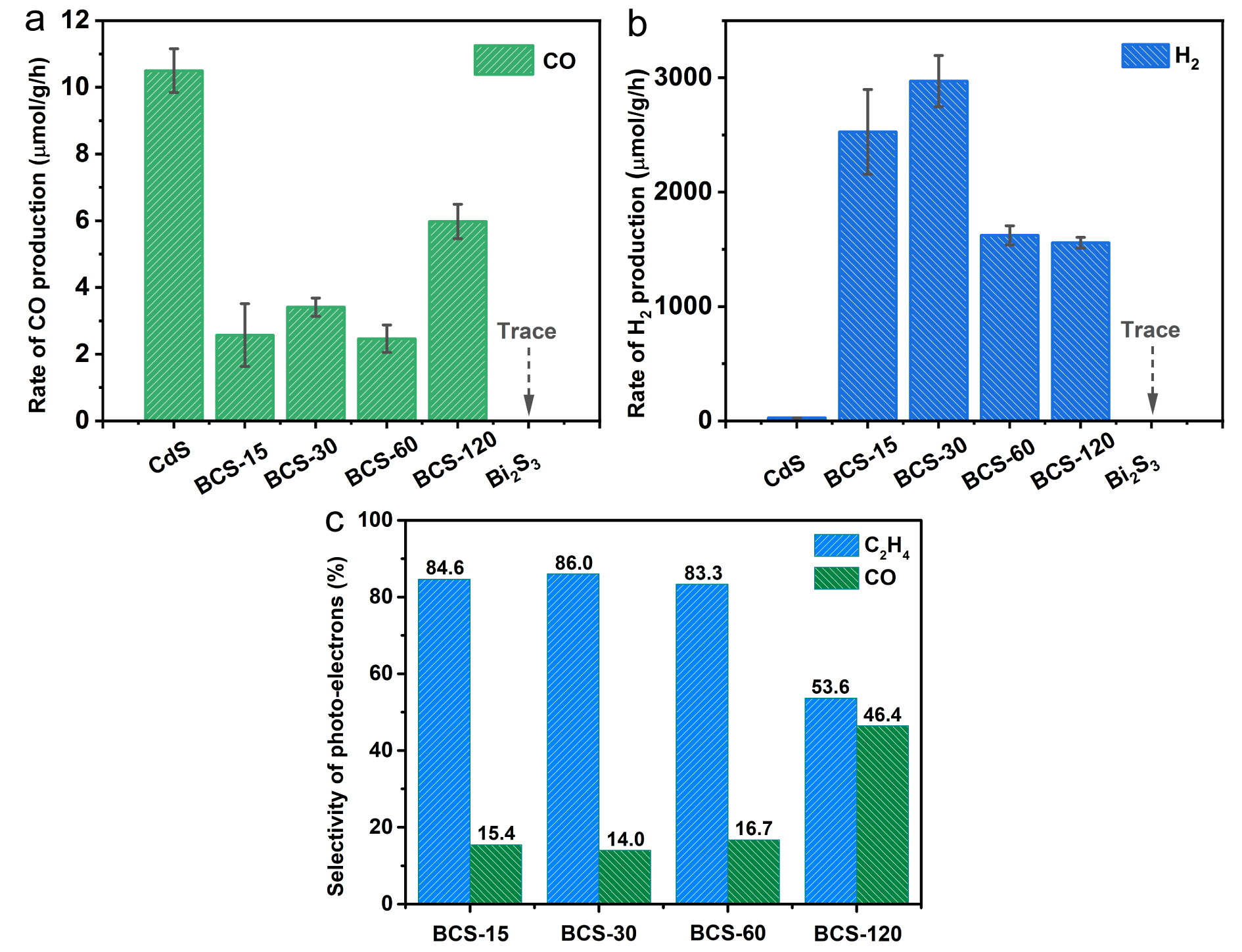


**Figure S8.** Long–term photocatalytic activity of (a) C_2_H_4_, (b) CO and (c) H_2_ production over the BCS–30; and (d) PXRD patterns of the BCS–30 before and after the long–term photocatalytic experiment.


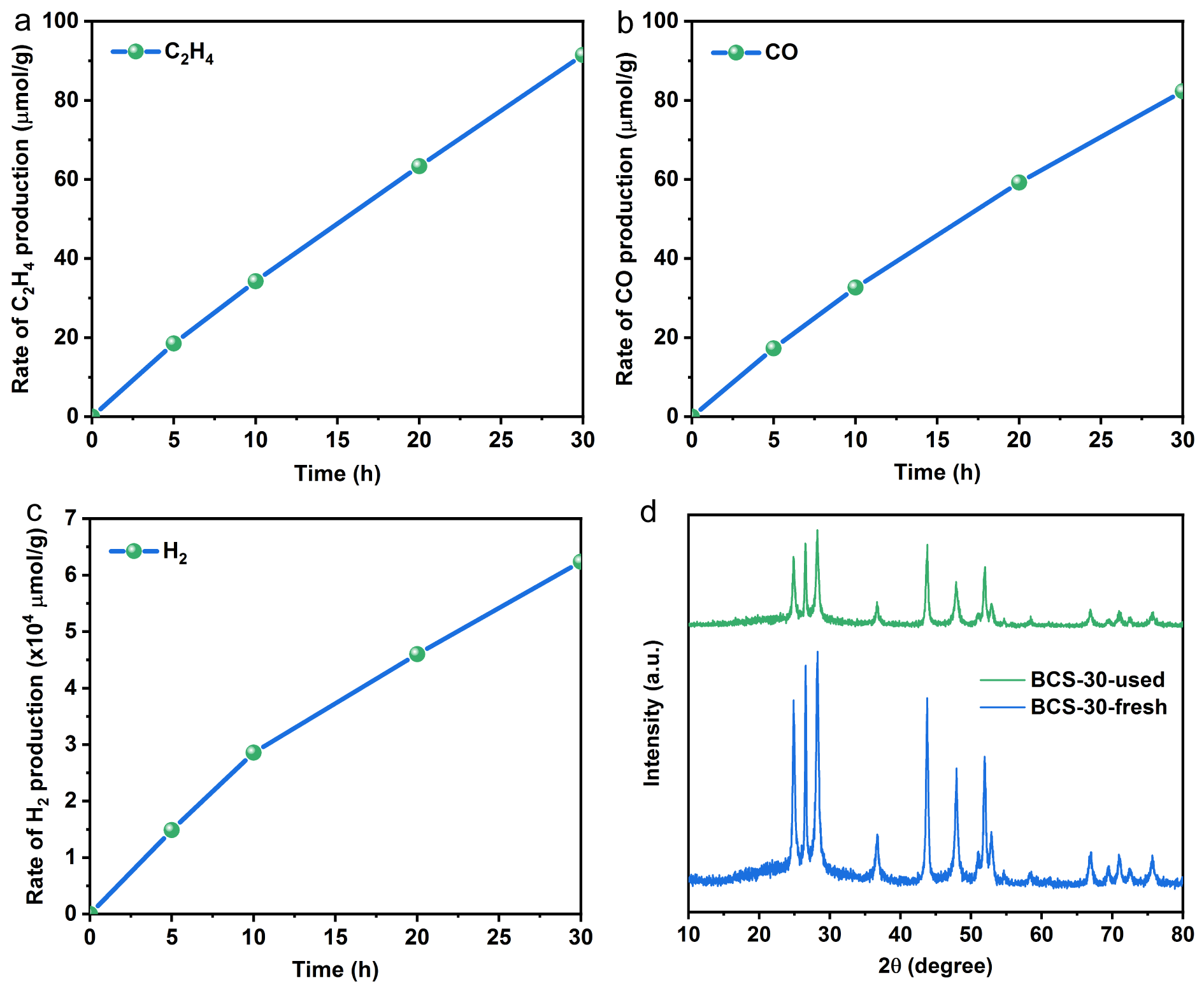


**Figure S9** (a) TEM, (b) HR–TEM, and (c) elemental mapping analyses of the BCS–30 before and after the long–term photocatalytic experiment.

**
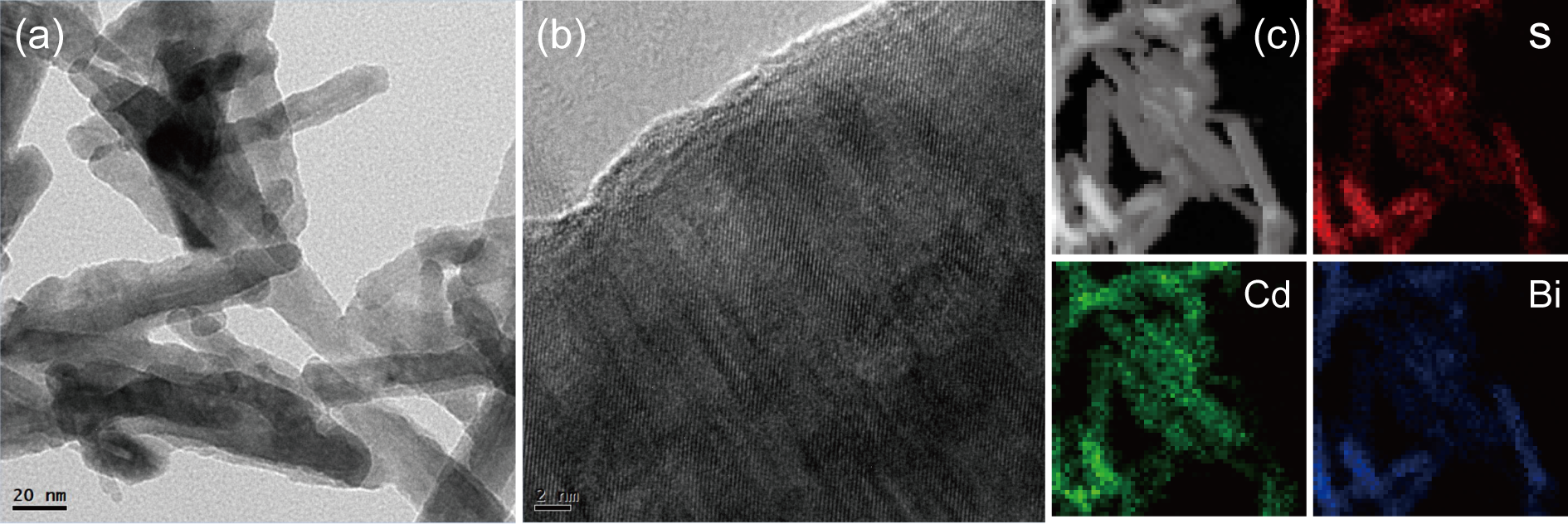
**

**Figure S10** (a) XPS full spectra XPS spectra of (b) Cd 3d, and (c) Bi 4f and S2p of the BCS–30 before and after the long–term photocatalytic experiment.

**
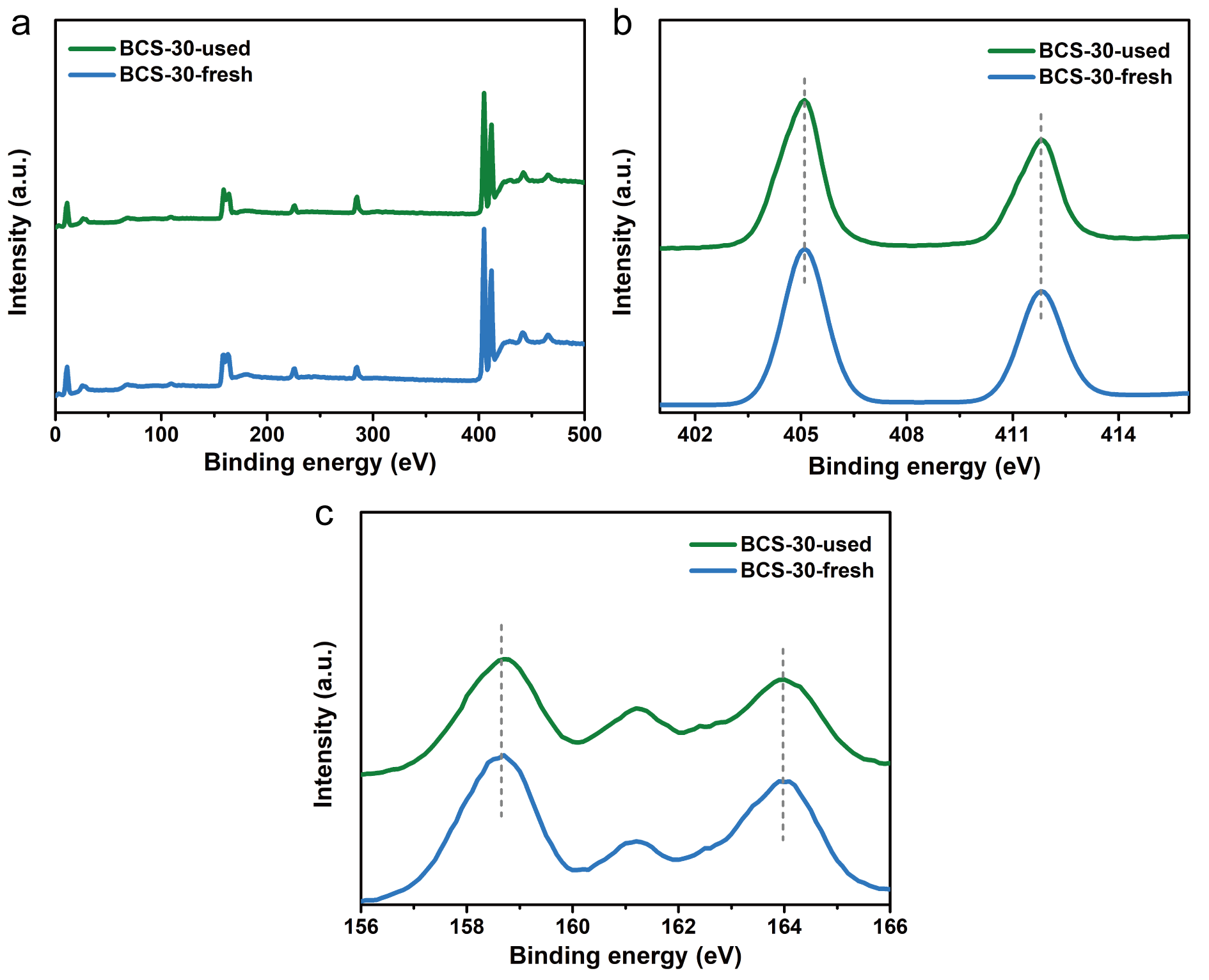
**

**Figure S11.** Photocatalytic activity (a) H_2_, (b) CO and (c) C_2_H_4_ production (NNS = Na_2_S/Na_2_SO_3_ aqueous solution) over the BCS–30; and (d) comparison of ethylene activity in different gas atmosphere (inset: H_2_ production rate).

**
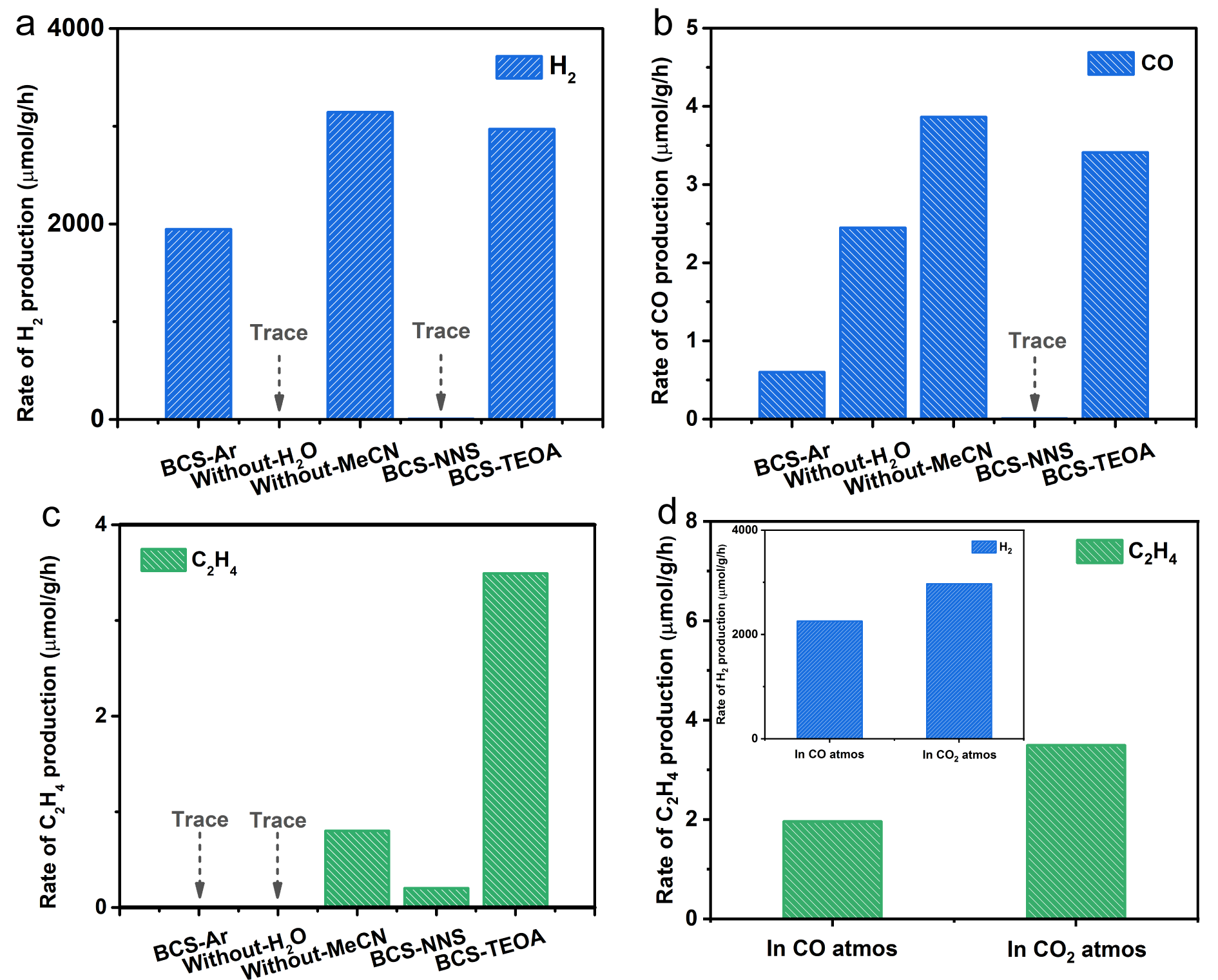
**

**Figure S12.** (a) Room temperature PL and (b) electrochemical impedance spectra of CdS, Bi_2_S_3_ and BCS–t.


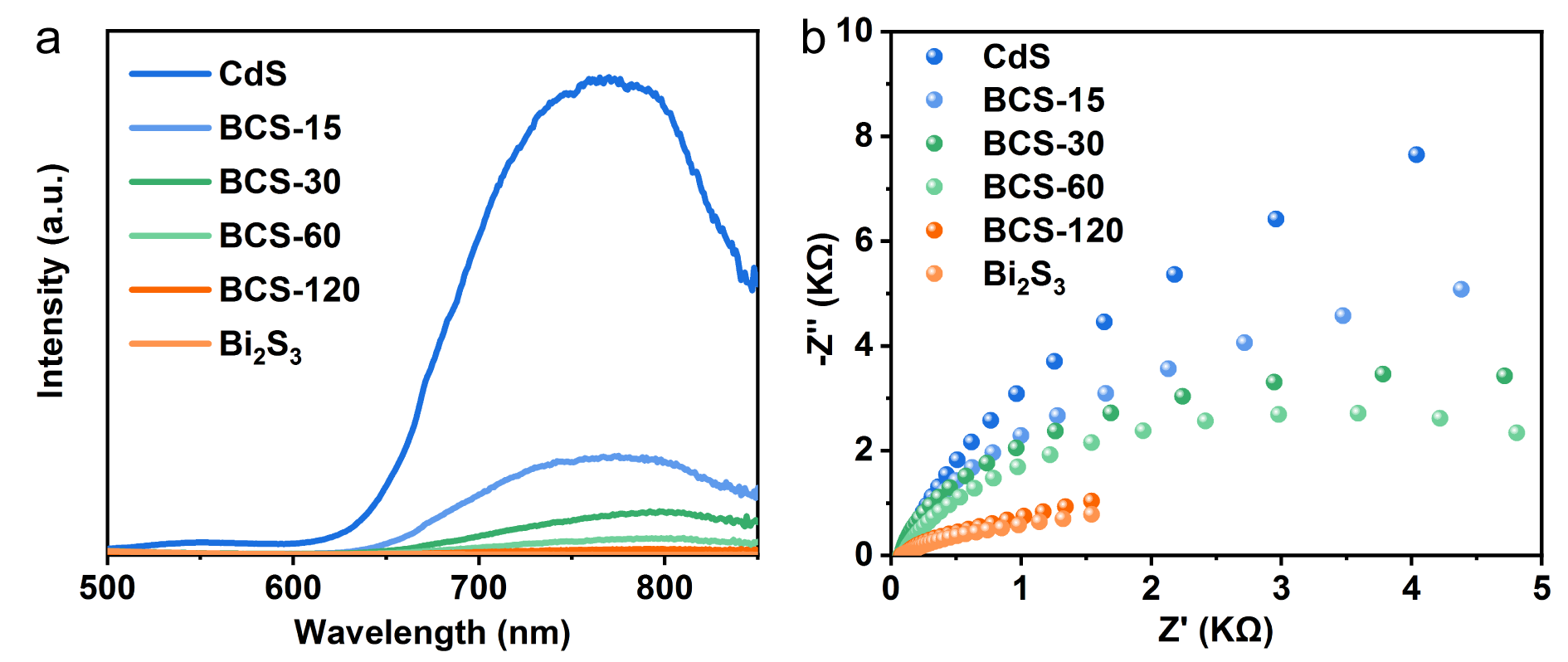


**Figure S13.** *In situ* DRIFTS for CdS in CO_2_ atmosphere under visible light irradiation.

**
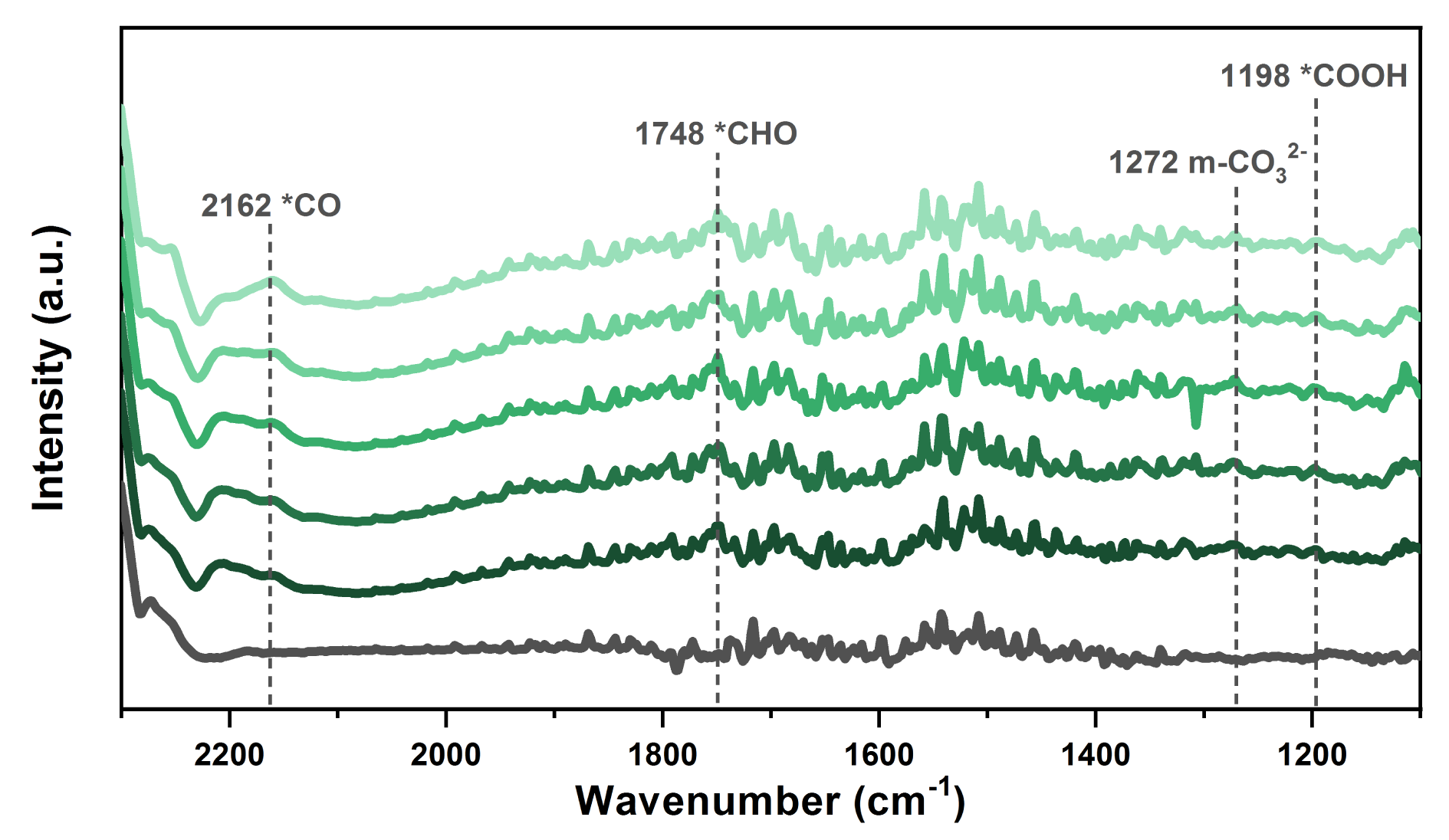
**

**Figure S14.** Structural evolution of intermediates in the CH_4_ pathway on the BCS surface.


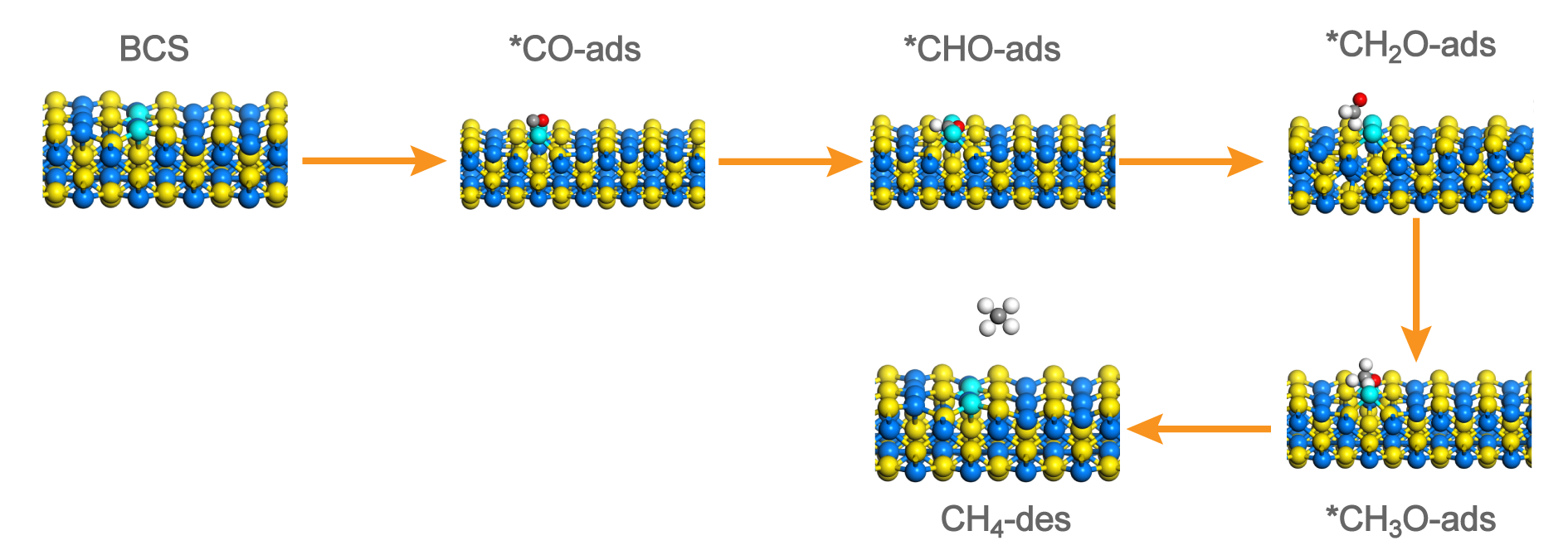


**Figure S15.** The free energy diagram of the various pathways of CO_2_–to–C_2_H_4_ photoreduction on the BCS at U = 0 V.


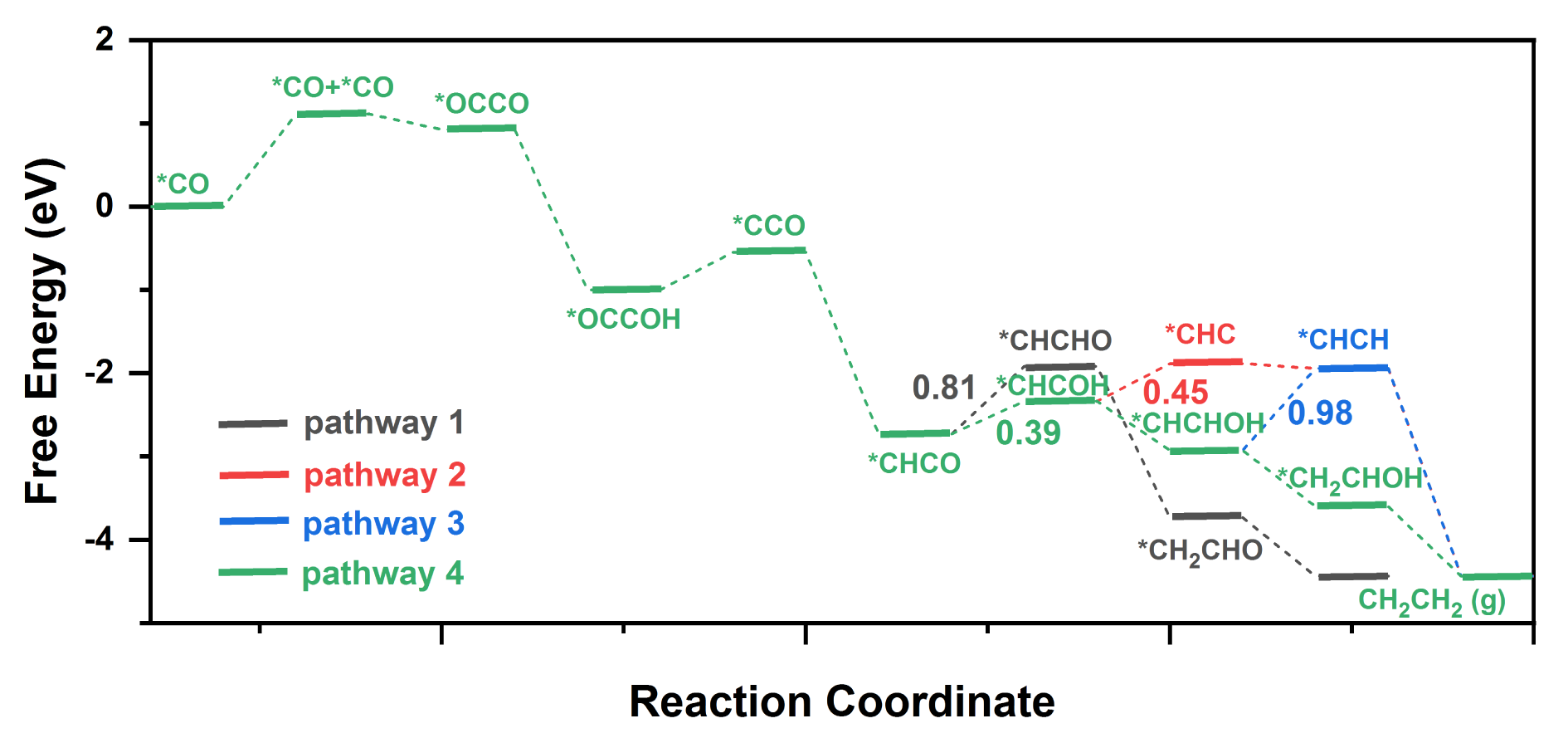


**Figure S16.** The structures of intermediates in various C_2_H_4_ production pathways on the BCS surface. (a) the CH_2_CHO pathway and (b) the CHC pathway.


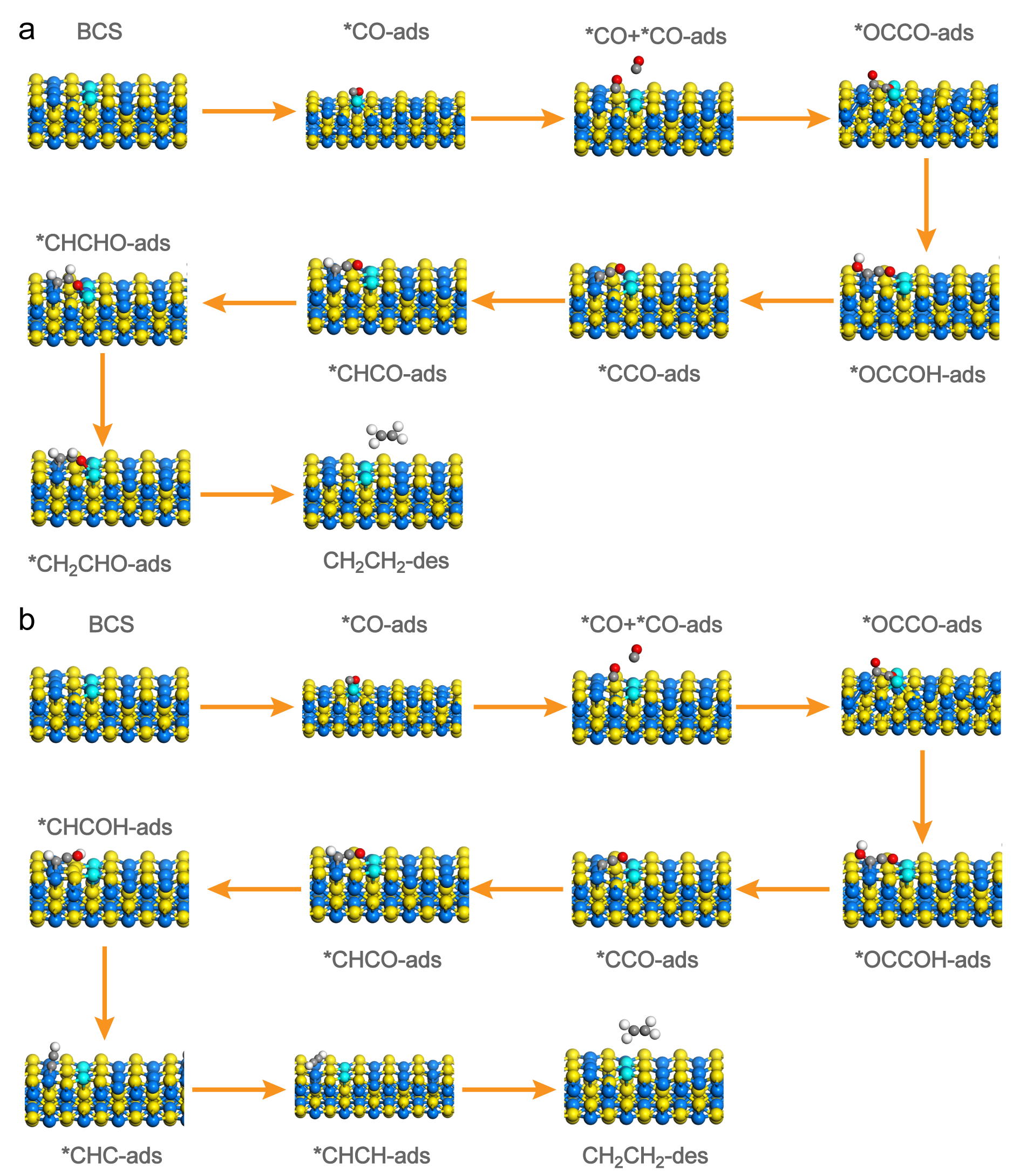

Supplement: Supplementary Materials — Table S1: elemental analysis of BCS–t heterostructures. Figure S1: PXRD patterns of the CdS and BCS–t. Figure S2: SEM images of (a) CdS and (b) BCS–30. Figure S3: (a) TEM and (b) HR–TEM images of the CdS. Figure S4: (a) and (b) TEM, (c) HR–TEM, and (d–h) elemental distribution images of the BCS–30. Figure S5: (a) UV–vis DRS and (b) K–M plots of CdS; (c) UV–vis DRS and (d) K–M plots of Bi2S3. Figure S6: Mott–Schottky plots of (a) CdS and (b) Bi2S3. Figure S7: yields of (a) H2 and (b) CO in CO2 photoreduction over CdS and BCS–t under visible irradiation. Figure S8: long-term experimental photocatalytic activity of (a) C2H4, (b) CO, (c) H2 of the BCS–30, and (d) PXRD patterns of the BCS–30 before and after the long-term photocatalytic experiment. Figure S9: (a) TEM, (b) HR–TEM, and (c) elemental distribution images of the BCS–30 before and after the long-term photocatalytic experiment. Figure S10: (a) XPS full spectra, XPS spectra of (b) Cd 3d and (c) Bi 4f and S2p of the BCS–30 before and after the long-term photocatalytic experiment. Figure S11: photocatalytic activity of (a) H2, (b) CO, (c) C2H4 (NNS=Na2S/Na2SO3 aqueous solution) of the BCS–30, and (d) comparison of ethylene activity in different gas atmosphere (inset: H2 production rate). Figure S12: (a) room temperature PL and (b) electrochemical impedance spectra of CdS, Bi2S3, and BCS–t. Figure S13: in situ DRIFTS for CdS in CO2 atmosphere under visible light irradiation. Figure S14: structural evolution of intermediates in the CH4 pathway on the BCS surface. Figure S15: the free energy diagram of the various pathways of CO2–to–C2H4 photoreduction on the BCS at U=0V. Figure S16: the structures of intermediates in various C2H4 production pathways on the BCS surface. (a) The CH2CHO pathway and (b) the CHC pathway. [file 9805879.f1.doc]
